# Monoamine oxidase A: An emerging therapeutic target in prostate cancer

**DOI:** 10.3389/fonc.2023.1137050

**Published:** 2023-02-13

**Authors:** Chia-Hui Chen, Boyang Jason Wu

**Affiliations:** Department of Pharmaceutical Sciences, College of Pharmacy and Pharmaceutical Sciences, Washington State University, Spokane, WA, United States

**Keywords:** monoamine oxidase A, prostate cancer, metastasis, castration resistance, antiandrogen therapy, tumor-stromal interaction

## Abstract

Monoamine oxidase A (MAOA), a mitochondrial enzyme degrading biogenic and dietary amines, has been studied in the contexts of neuropsychiatry and neurological disorders for decades, but its importance in oncology, as best exemplified in prostate cancer (PC) to date, was only realized recently. PC is the most commonly diagnosed non-skin cancer and the second deadliest malignancy for men in the United States. In PC, the increased expression level of MAOA is correlated with dedifferentiated tissue microarchitecture and a worse prognosis. A wealth of literature has demonstrated that MAOA promotes growth, metastasis, stemness and therapy resistance in PC, mainly by increasing oxidative stress, augmenting hypoxia, inducing epithelial-to-mesenchymal transition, and activating the downstream principal transcription factor Twist1-dictated multiple context-dependent signaling cascades. Cancer-cell-derived MAOA also enables cancer-stromal cell interaction involving bone stromal cells and nerve cells by secretion of Hedgehog and class 3 semaphorin molecules respectively to modulate the tumor microenvironment in favor of invasion and metastasis. Further, MAOA in prostate stromal cells promotes PC tumorigenesis and stemness. Current studies suggest that MAOA functions in PC in both cell autonomous and non-autonomous manners. Importantly, clinically available monoamine oxidase inhibitors have shown promising results against PC in preclinical models and clinical trials, providing a great opportunity to repurpose them as a PC therapy. Here, we summarize recent advances in our understanding of MAOA roles and mechanisms in PC, present several MAOA-targeted strategies that have been nominated for treating PC, and discuss the unknowns of MAOA function and targeting in PC for future exploration.

## Introduction

Monoamine oxidase A (MAOA) is a flavoprotein that catalyzes the oxidative deamination of a number of biogenic and dietary amines, generating hydrogen peroxide, a major source of reactive oxygen species (ROS), as a by-product in the process. It is anchored to the mitochondrial outer membrane through a single transmembrane helix at the C-terminus with the N-terminus facing the cytoplasm. Many endogenous monoamines are neurotransmitters, such as serotonin and norepinephrine, and as a result MAOA has been most studied in the nervous system so far (1). This X-linked gene is nicknamed the “warrior gene” because of its association with aggressive behaviors in males (2, 3). Insufficient MAOA expression predisposes children to violence and antisocial behaviors if they experience maltreatment (4, 5). MAOA levels in the brain peak during early development and then remain relatively stable throughout life (6). Monoamine oxidase inhibitors (MAOIs) have been developed and used clinically to treat depression for decades (7).

After considerable interest and effort focused on understanding the role of MAOA in the brain, recent studies have started to reveal the importance of MAOA in cancer. A growing body of evidence indicates that MAOA either has altered expression levels or exerts a regulatory effect in a variety of types of cancer. Prostate cancer (PC) is the most well studied cancer type in this context (8–14). PC is the most frequently diagnosed non-cutaneous malignancy and second leading cause of cancer death in men in the United States (15). MAOA has been consistently found to be highly expressed in PC and to promote cancer growth, progression and metastasis (14, 16). Consequently, inhibition of MAOA by repurposing clinically available MAOIs has become an enticing strategy for treating PC, as demonstrated in both preclinical models and clinical trials to date (16–18). This review summarizes our current knowledge of MAOA in PC, including its clinical relevance, function, mechanism and targeting potential, and we also discuss MAOA’s remaining unknowns for future studies.

## MAOA expression is correlated with PC risk, progression and prognosis

In an attempt to molecularly characterize PC according to Gleason grades, which histologically annotate cancers based on microscopic tumor architecture, True and colleagues presented the first evidence that MAOA expression is progressively elevated along with increasing Gleason grades representing less differentiated morphology in PC patient samples (14), thus implicating MAOA’s contributory role in PC progression. This observation was reproduced in other follow-up studies, including ours, where MAOA was found to be upregulated in high Gleason-grade prostate tumors (16, 19). MAOA expression level is also positively associated with preoperative serum prostate specific antigen (PSA), which is a classical diagnostic and prognostic factor in PC. Subsequently, Zhao and colleagues showed that inhibition of MAOA with clorgyline, an irreversible MAOA inhibitor, promoted secretory differentiation in cultured human primary basal prostate epithelial cells (20), consistent with the observation that MAOA is associated with the de-differentiated morphology of high-grade PC. Later, we demonstrated in experimental models that MAOA is upregulated at the molecular level under the orchestrated control of aberrant oncogenic (activation of c-Myc and loss of PTEN and p53) and androgenic signaling during the disease trajectory (16), which coincides with the MAOA expression pattern observed in clinical samples. The promoter region of the *MAOA* gene contains between two and five copies of a 30-nucleotide variable number tandem repeat (VNTR), whose copy number has been linked to different MAOA expression levels (21). In a study with approximately 1,300 Caucasian participants each in both the PC and control groups, a five-copy low-expression *MAOA* VNTR polymorphism was associated with a lower risk of developing PC (22). In addition, we interrogated multiple independent patient cohorts and revealed that high MAOA expression is associated with poor prognosis as evidenced by several clinical parameters including seminal vesical invasion, biochemical recurrence, metastasis and survival (16). Collectively, these earlier clinically relevant studies suggested that MAOA may have a previously unrecognized function in PC meriting further research.

## MAOA promotes growth and metastasis of PC cells

MAOA has been increasingly recognized for the ability to promote the growth and metastasis of PC cells in a variety of preclinical models. Overexpression of MAOA in PC-3 cells accelerated cancer cell proliferation, migration and invasion and tumor formation and growth, while genetic knockdown or pharmacological inhibition of MAOA reduced these aggressive behaviors in multiple human (LNCaP, C4-2 and ARCaP_M_) and mouse (MPC3 bearing a double knockout of *Pten* and *Tp53*) PC cell lines *in vitro* and *in vivo* (16). Flamand and colleagues reported that treating VCaP cells with clorgyline also inhibited cell growth both in cultures and in a mouse xenograft model (18). In an intracardiac xenograft model that rapidly develops distant metastasis to bone and visceral organs through the bloodstream, overexpression of MAOA in the bone-tropic PC-3 cells promoted a higher incidence of metastasis and greater skeletal tumor burden, with more circulating tumor cells detected in peripheral blood at the endpoint, suggesting the heightened potential of metastatic seeding for further dissemination. Conversely, knockdown of MAOA or pharmacological inhibition of MAOA delayed the onset of metastasis, reduced tumor burden in bone and visceral organs, and prolonged mouse survival compared to controls (23). Mechanistically, MAOA stabilizes HIF1α by the generation of ROS and destruction of PHD3, an oxygen-dependent prolyl hydroxylase rendering HIF1α degradation. Increased HIF1α transcriptional activity in turn activates VEGF-A/neuropilin-1 (NRP1) signaling to upregulate the AKT/FOXO1 pathway, which results in nuclear export of the transcription repressor FOXO1 to induce Twist1-dictated epithelial-to-mesenchymal transition (EMT) and paracrine sonic hedgehog (Shh) signaling, creating a vicious cycle between cancer and stromal cells (16, 23). These constitute a MAOA-directed interplay between oxidative stress, hypoxia, EMT and tumor-stromal interaction to cooperatively promote cancer cell aggressiveness and invasiveness. Interestingly, the MAOA/HIF1α signaling pathway was later found to be responsible for EMT induced by cancer-associated fibroblasts in PC as well (24). In addition to nurturing blood-borne metastasis, we recently showed that MAOA also promotes perineural invasion, a nerve-facilitating route of PC metastasis, in a 3-dimensional cancer-nerve cell co-culture model, and tumor innervation in an orthotopic xenograft model. MAOA mediates this process by activating semaphorin 3C (SEMA3C) in a Twist1-dependent transcriptional manner, which in turn stimulates cMET to confer neoplastic invasion of nerves *via* autocrine or paracrine interaction with coactivated PlexinA2 and NRP1, the receptor and co-receptor of SEMA3C respectively (25). Using a spontaneous prostate tumor mouse model with prostate-specific knockout of *Pten* and *Maoa*, Liao and colleagues further showed that ablating MAOA in the prostate epithelial cells reduced the development and inhibited the growth of prostate adenocarcinoma with decreased cancer stem cell population. Concordantly, clorgyline was found to be less effective in inhibiting the growth and stemness of PTEN-positive 22Rv1 cells than PTEN-negative LNCaP cells, showing that MAOA’s functions in PC depend on the PTEN status (26). These studies in aggregate demonstrate the essential role of MAOA in mediating PC development, growth and metastasis.

## MAOA confers castration resistance in PC

PC depends exquisitely on androgen receptor (AR) activity for growth, survival and progression, making AR-targeted therapy a mainstay treatment for PC for decades. However, PC inevitably relapses after therapy escape and progresses to fatal castration-resistant prostate cancer (CRPC) with near-universal reactivation of AR signaling (27). Thus, understanding the mechanisms for the emergence of castration resistance has been a central focus in the PC field. MAOA was first predicted as an androgen-regulated gene in LNCaP cells in 2004 (28). Later, using an *in vivo* LNCaP Hollow Fiber model, Romanuik and colleagues generated three groups of LNCaP cells representing different stages of hormonal progression that were androgen-sensitive (AS), responsive to androgen-deprivation (RAD, 10 days after castration), and castration-recurrent (CR, 72 days after castration). Their results showed that *MAOA* mRNA expression decreased in the RAD group but partially recovered in the CR group (29), suggesting that *MAOA* was transcriptionally upregulated under a castration-resistant state and could be important for developing castration resistance. Indeed, AR binds to the promoter and 5’-UTR of *MAOA* to activate its expression under low-androgen conditions and supports the androgen-independent growth of androgen-dependent LNCaP cells (30). Subsequently, our group identified a novel androgen response element in intron 3 of the *MAOA* gene, which is bound by AR to induce *MAOA* transcription, and detailed a reciprocal regulatory mechanism between AR and MAOA. In addition to AR transactivation of MAOA, we showed that MAOA promotes the transcriptional activity of AR by upregulating its co-activator YAP1 for enhanced nuclear YAP1-AR interaction, forming a positive feedback loop in both androgen-dependent and CRPC cells. MAOA induces YAP1 through downstream ROS/Twist1-dependent activation of Shh/Gli signaling for direct Gli1/2 interaction with a Gli-binding site in *YAP1* promoter (31). Of note, MAOA expression is elevated and associated with AR activity in CRPC clinical specimens (31, 32). Importantly, knocking down MAOA expression impeded tumor growth of both androgen-dependent LNCaP cells and castration-resistant C4-2B^ENZR^ cells (a LNCaP-derived CRPC subline with acquired resistance to the antiandrogen drug enzalutamide) (31), indicating that MAOA is a valid target for AR-positive PC cells regardless of cellular reliance on androgens. Our findings are consistent with a previous study showing that MAOIs decrease proliferation of both androgen-responsive and CRPC cells and that clorgyline represses both the full-length AR and AR-V7 (33), a clinically relevant splicing variant of AR that lacks a ligand binding domain and possesses constitutive activity (34). Utilizing glucocorticoids either as an alternative ligand to activate AR target genes through a mutated AR (35) or as a cognate ligand to activate the glucocorticoid receptor (GR) as an AR substitute (36) is a well-accepted mechanism conferring castration resistance. Interestingly, MAOA was recently reported to be also upregulated by GR through direct GR binding at a region in intron 3 of the *MAOA* gene for transcriptional induction in PC cells (37), once again suggesting its relevance to the development of resistance to castration and antiandrogen therapy. Further, MAOA was shown to be activated by relieving REST transcriptional suppression to inhibit apoptosis while activating autophagy to induce the neuroendocrine differentiation of LNCaP cells upon androgen deprivation (38), where acquisition of neuroendocrine features is an emerging mechanism enabling AR-driven prostate adenocarcinoma to evolve into AR-indifferent CRPC with resulting antiandrogen resistance (39, 40). MAOA was also found to maintain the neuroendocrine differentiation of C4-2 and 22Rv1 CRPC cells upon short- or long-term exposure to enzalutamide (41). Together, these studies emphasize the importance of MAOA in developing resistance to castration and antiandrogen drugs in PC cells, making it a potential target for preventing or overcoming treatment resistance in lethal PC.

## MAOA modifies the tumor microenvironment in favor of cancer progression and metastasis

MAOA promotes PC metastasis not only by promoting EMT in cancer cells *per se* but also by modifying the tumor microenvironment. Tumor-derived MAOA stimulates osteoclastogenesis, tipping the balance towards bone destruction and creating a pre-metastatic niche for initiating bone metastasis. Pharmacological inhibition of MAOA with clorgyline significantly decreased both the number of osteoclasts and the osteolytic area in the hind limb of mice intracardiacally injected with PC-3 cells. MAOA induces osteoclastogenesis by triggering the production and release of IL-6 and RANKL from osteoblasts, which is dependent on the transcription factors Gli1 and Gli2 expressed in osteoblasts while activated by Shh molecules secreted from tumor cells in the presence of MAOA (23). In addition, MAOA expression is elevated in the prostate stromal cells associated with cancer relative to normal counterparts, which reprograms the stroma to a more reactive and pro-tumorigenic state through increased oxidative stress to promote PC development, growth and stemness. Knocking down MAOA in human prostate stromal fibroblast PrSC cells suppressed the growth, migration, invasion, and stem-like properties of multiple human PC cell lines either co-cultured with stromal cells in a direct-contact manner or exposed to stromal cell conditioned media. Co-inoculating MAOA-depleted PrSC cells with PC-3 cells and treating mice growing PC-3 cells with clorgyline in a stromal-specific targeted manner resulted in smaller tumors in a subrenal capsule xenograft model and in an orthotopic xenograft model respectively compared to controls. Mechanistically, MAOA in stromal cells enhances *IL-6* transcription and expression through Twist1 binding to an E-box element on the *IL-6* promoter, which in turn activates paracrine IL-6/STAT3 signaling to transcriptionally upregulate the cancer stem cell marker CD44 in adjacent PC cells (42). Collectively, these studies demonstrate that MAOA of tumor or stromal cell origins dictates tumor-stromal cell interactions to favor reprogramming of naïve stroma towards a tumor-supportive phenotype.

## The therapeutic potential of MAOIs in PC

Given that MAOA promotes PC progression and that its inhibitors are already used in the clinic, MAOA has become a promising target for developing treatment strategies against PC. MAOA also possesses a unique advantage as a therapeutic target given its dual targeting potential in both the tumor and stromal components to effectively block tumor progression (16, 42). Interim results from a recent Phase 2 clinical trial (ClinicalTrials.gov Identifier: NCT02217709) have shown that phenelzine, a non-selective MAO inhibitor, demonstrates efficacy as serum PSA declines in patients with biochemical recurrent castration-sensitive PC (17). In addition to use as a single therapy, MAOIs also offer synergistic effects when combined with other treatments. Clorgyline and phenelzine enhance the growth inhibition effects of enzalutamide, darolutamide and apalutamide, all second-generation antiandrogen drugs currently used clinically to treat CRPC (43), in androgen-dependent LNCaP and LNCaP-derived CRPC (C4-2 and LNCaP-abl) cells (31, 37). Clorgyline and phenelzine have also been shown to reverse the enzalutamide resistance of resistant CRPC cells (C4-2 and 22Rv1) in cultures and in a mouse xenograft model (44). Moreover, combined treatment of naïve or antiandrogen-resistant PC cells with clorgyline and chemotherapeutic agents, including docetaxel and cabazitaxel, resulted in more reductions in cell growth compared to chemotherapy alone (37, 45). Further, Xu and colleagues reported that dual pharmacological inhibition of MAOA and survivin produced significant synergy in inhibiting the proliferation, migration and invasion of PTEN-negative PC cells (46). Interestingly, while it has been shown that ERG-positive PC cells tend to express MAOA at a relatively lower level compared to ERG-positive cells (47), the fact that clorgyline inhibits the growth of both ERG-positive VCaP and ERG-negative LNCaP cells (48) suggests that MAOA could be a valid target in multiple types of PC even with varied levels of MAOA. In addition to repurposing clinically available MAOIs, a novel MAOA inhibitor – NMI – was synthesized by conjugating a tumor-targeting near-infrared dye to clorgyline, demonstrating enhanced effectiveness in suppressing prostate tumor growth compared to clorgyline and also providing a good imaging tool for prognostic purposes (49). Further, there is a growing interest in exploring the utility of natural MAOIs (24, 50) and using them as the basis to develop new MAOIs as cancer therapeutics for clinical applications (51, 52). Despite these advances, it is also important to note that the effectiveness of MAOA inhibitors could be dissimilar as tested in different PC cell lines, indicating the need for a better understanding of MAOA’s role in different genomic and cellular contexts as well as careful stratifications of patients by molecular biomarkers to achieve precise application of MAOIs with maximal efficacy. Together, these studies strongly support repurposing MAOIs alone or in combination with other existing therapies as an attractive treatment strategy for PC, which merits further development, validation and optimization to move forward into clinical use.

## Conclusion and future directions

**Figure 1:**
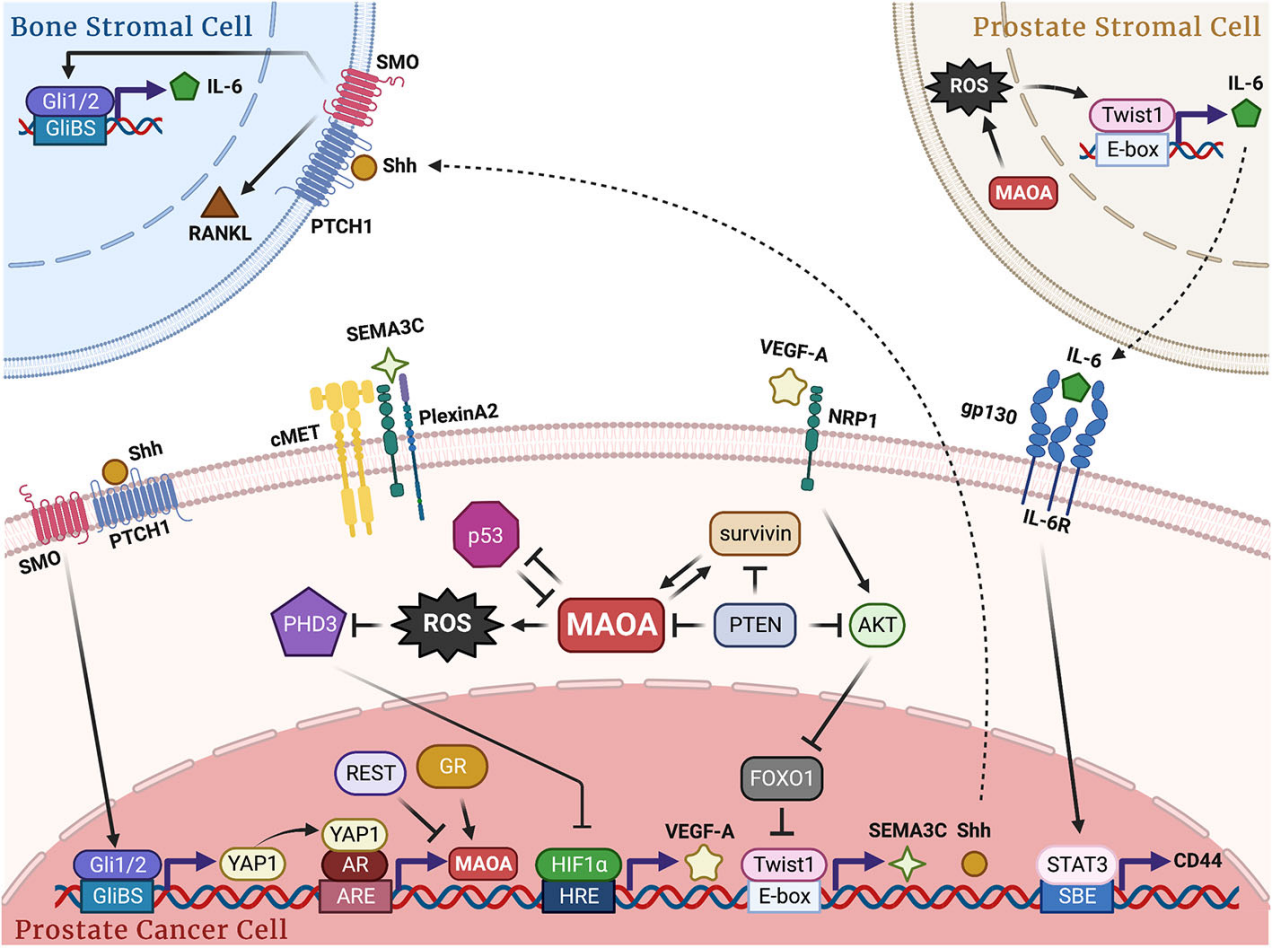
Schematic of the molecular mechanisms by which MAOA promotes PC growth, progression and metastasis. In PC cells, MAOA generates ROS to repress PHD3, which stabilizes HIF1α and induces VEGF-A/NRP1 signaling to upregulate the AKT/FOXO1 pathway, resulting in activation of Twist1 by relieving FOXO1 transcriptional suppression with subsequent induction of Shh and SEMA3C in a Twist1-dependent transcriptional manner. Next, Shh interacts with PTCH1 to relieve depression of SMO and activate Gli1 and Gli2, which transcriptionally induces YAP1 to enhance YAP1-AR interaction, promoting AR transcriptional activity and fueling AR-reliant cancer growth. SEMA3C interacts with PlexinA2 and NRP1 to stimulate cMET for perineural invasion. Upstream regulators AR, GR, REST, PTEN, p53 and survivin cooperatively contribute to *MAOA* gene regulation either in a known transcriptional manner or through an unidentified mechanism. In cancer-bone stromal cell crosstalk, MAOA-expressing cancer cells secrete Shh *via* PTCH1/SMO to activate Gli1 and Gli2 in osteoblasts in a paracrine manner, which in turn induces the expression and release of IL-6 and RANKL from osteoblasts to promote osteoclastogenesis and subsequent bone metastasis. In prostate stromal-cancer cell crosstalk, MAOA in stromal fibroblast cells triggers *IL-6* transcription and expression in a ROS/Twist1-dependent manner, which in turn interacts with gp130/IL-6R expressed in cancer cells for STAT3-dictated transcriptional activation of CD44 to confer cancer stemness. GliBS, Gli-binding site; ARE, androgen response element; HRE, hypoxia response element; SBE, STAT-binding element.

Overall, it has been established that high expression levels of MAOA correlate with worse prognosis and that MAOA promotes growth, progression, metastasis and therapy resistance in PC, which provides a strong clinically relevant rationale for targeting MAOA and repurposing MAOA inhibitors for treating PC. Recent studies have greatly expanded our understanding of PC disease mechanisms at the molecular level, where MAOA primarily utilizes the ROS-Twist1 axis as a central downstream mediator to trigger multiple cellular and molecular events, including cancer-cell-intrinsic signaling cascades, cancer cell crosstalk with the tumor microenvironment, and stromal reprogramming, to collaboratively foster PC development and progression (**Figure 1**). Although MAOA has been extensively studied in recent years, there are still some unknowns remaining to be explored concerning the role, mechanism and targeting of this fascinating molecule in PC. In addition to ROS, other molecules generated in the oxidative deamination reaction have received little attention. For example, ammonia is normally considered a toxic waste processed in the liver into urea and excreted. However, many types of cancer express elevated levels of ammonia-assimilating enzymes. Breast cancer has been demonstrated to recycle ammonia as the nitrogen source to support tumor growth (53). It is thus tempting to speculate that ammonia produced by MAOA may enter a recycling process to promote growth in PC as well, which is a likely mechanism given that HIF1α can be stabilized by ammonia in cancer and appears to be a well-conserved effector in ammonia stress (54–56). Immunotherapy has shown major success in treating several types of cancer, but it has very limited benefit in PC due to PC’s complex immunosuppressive tumor microenvironment (57). Recent studies have shown that immune-cell-derived MAOA promotes an immunosuppressive tumor microenvironment by disrupting T cell and macrophage differentiation in mice, and that combining MAOI and anti-PD-1 treatments resulted in synergistic tumor suppression in melanoma and colon cancer (58, 59). However, to our knowledge, no studies examining the relationship between MAOA, especially cancer-cell-intrinsic MAOA, and antitumor immunity in PC have been published to date, which certainly warrants further studies to potentially extend the utility of MAOIs in combination with immunotherapy to combat PC with improved efficacy. Lastly, emerging studies, including ours, have demonstrated that high cellular and epigenetic plasticity is a major mechanism of therapy resistance and progression to lethal stages such as metastatic castration-resistant and neuroendocrine PCs (40, 60). Despite the known association of MAOA with neuroendocrine differentiation (38, 41), whether and how MAOA contributes to acquisition of lineage plasticity to transform cancer cells from a luminal epithelial phenotype into a more dynamic state integrating properties of other lineages, such as basal, neuroendocrine, neural and mesenchymal lineages, under selective therapeutic pressures remains largely unclear, and merits future studies to provide MAOA-targeted therapeutic strategies to treat lethal PC.

## Author contributions

C-HC performed the literature search, designed the figure, and wrote the original draft. BW reviewed and edited the figure and manuscript. Both authors contributed to the article and approved the submitted version.
